# The Treatment of the Pedicle in Ovariotomy

**Published:** 1876-09

**Authors:** Frederick W. Strange

**Affiliations:** M. R. C. S., Eng., Fellow Obstetrical Society, London


BUFFALO

### Medical and Surgical Journal.

VOL XVI. SEPTEMBER, 1876. No. 2.

### Original Communications.

:o:

ART. I.—*The Treatment of the Pedicle in Ovariotomy.* A paper read before the Canadian Medical Association at Toronto, Ont., August 2d, 1876. By FREDERICK W. STRANGE, M. R. C. S. Eng., Fellow Obstetrical Society, London.

MR. PRESIDENT AND GENTLEMEN:—In view of the fact that the operation of ovariotomy is now recognized by all Surgeons as not only a justifiable operation in suitable cases, but absolutely imperative for prolonging to the natural period the life of the unfortunate sufferer from ovarian disease, and as the treatment of the pedicle is the only point of difference existing in the mode of operation in the hands of different surgeons, I have ventured to bring before your notice a brief comparative synopsis of the various methods employed in treating the pedicle after the tumor has been removed.

For the sake of brevity I shall divide the various forms of treatment into two classes, the first embracing the modes of treatment by leaving the extremity of the pedicle outside of the abdominal walls; the second embracing the modes of treatment when the pedicle is returned into the pelvic cavity.

In the first class, the pedicle may be secured outside the abdominal walls by Spencer Wells' clamp, by callipers of various forms, or by transfixing the end of the pedicle with a needle, which by being passed through the lower parts of the abdominal incision and transfixing the pedicle between the walls, does the double duty of preventing the return of the pedicle, and at the same time closing the lower part of the abdominal incision.

The rationale of these plans is the same whatever method is employed, viz: the arrest of hemorrhage from the pedicle by pressure, and the prevention of its return within the abdomen.

This mode of treatment possesses great advantages in those cases in which the pedicle is long and the vessels of the pedicle large, since it insures all safety, so far as the pedicle is concerned, against internal hemorrhage and suppuration; but it labors under the disadvantage that when the pedicle is short it is difficult of application, it pulls the uterus out of place, it disturbs the pelvic organs, and frequently gives rise subsequently to uncomfortable dragging sensations in the lower portion of the abdomen and in the pelvis.

The second class of treatment in which the pedicle is returned into the pelvic cavity obviates these objections, but is more prone to the dangers of internal hemorrhage and suppuration. Cases imperatively demanding this method are those in which the pedicle is very short, and where the ovarian tumor is almost sessile, so to speak, to the uterus. The plan adopted by some surgeons in these cases, is to secure the pedicle by transfixing it with a strong silk or hempen ligature, or by silver wire, tying both halves tightly, cutting the ends off short, closing up the abdominal incision, and trusting to Providence that the ligature or wire may do no harm. The risks in this case are, first, that the ligature or wire may slip or become untied and fatal hemorrhage ensue; and secondly, that danger having been escaped, that their presence may excite inflammatory action; and thirdly, that the disintegration and sloughing of the constricted end of the pedicle may cause a collection of pus for which there is no egress.

Another plan of internal treatment is to secure the end of the pedicle by a strong ligature of silk hemp, or whipcord, and allow the end of the ligature to escape through the lower part of the abdominal incision. This plan is open to most of the objections just enumerated, but is a decided advance in treatment in this respect, that in case of internal collections of pus and debris from the extremity of the pedicle, the ligature serves as a guide for its discharge externally. The principal objection is that in some cases recovery is greatly retarded on account of the ligature remaining firmly attached to the pedicle for weeks or even months. This persistence is, I believe, due to the fact of too much tissue having been taken up by the ligatures or by their not having been drawn sufficiently tight. Time however ultimately triumphs, and the ligatures come away, but not before having caused considerable annoyance and delay, especially to those patients who have left their homes to be near their surgeons.

There remains another method which, I believe, will ultimately be adopted by surgeons as the safest, most rational, and at the same time the neatest way of treating the pedicle. I allude to the division by means of the actual cautery for which the profession is indebted to the late Mr. Baker Brown. As I have not met with the records of any case of ovariotomy performed in Canada, in which the pedicle was treated in this manner, I will briefly describe its application. As soon as the tumor has been brought fairly outside the abdominal incision, a clamp, which I shall have much pleasure in exhibiting in a few minutes, is applied to the pedicle. A cautery iron *heated just short of white heat*, is pressed backwards and forwards against the pedicle until it *gradually burns its way* through its thickness. The clamp is then removed *gradually*, care being taken to see that no hemorrhage occurs on its removal, and the pedicle is allowed to slip back into the pelvic cavity, and the abdominal incision is then closed.

The extremity of the pedicle when treated in this manner, shows no evidence of having been cauterized beyond a fine eschar which looks like a hair; there is no irritating ligature or wire to give rise to inflammatory action; there is no strangled extremity to slough and cause suppuration; while in more than one hundred cases actual experience has shown that there is not the least fear of secondary hemorrhage. If hemorrhage occurs at all, it will take place immediately the clamp is removed and while the pedicle still remains outside the abdomen, and if it does take place then, which is of very rare occurrence, it can be immediately arrested by a ligature.

Statistics are proving that the rate of mortality by this treatment is less than by any other. Of fifty consecutive cases treated in this way by the late Mr. Baker Brown, forty-five recovered, bringing the rate of mortality *to ten per cent.* More recently, Mr. Alexander Keith, of Edinburgh, has excelled even this brilliant record, having had forty-six recoveries out of fifty consecutive cases treated in this manner, thus bringing the rate of mortality down to *eight per cent.* Supported by this splendid success and backed by my own experience both during my own association with the late Mr. Baker Brown, and since his demise, I have every confidence in bringing the treatment of the pedicle by actual cautery under your notice to-day, and most heartily recommending its adoption by all ovariotomists in this country, recommended as it is by such distinguished surgeons as the late Mr. Baker Brown, Mr. Alexander Keith, and the late Dr. Tanner of London, England. The latter lamented gentleman in his "Practice of Medicine," 5th edition, published in 1865, says, when speaking on this subject, Mr. Baker Brown has recently resorted to the use of the actual cautery; and I cannot but think that if this plan works well on further repetition it will supersede all others.

Of the five unsuccessful cases in Mr. Baker Brown's series, I have the notes of the operation, and post mortem examinations of three which proved fatal in the London Sugical Home for women.

Case No. 1. M. R., aet 25, unmarried. Catamenia always regular. Her health had been perfectly good until three years ago when pain in the left side first was felt. Swelling has only been noticed for the last seven months. Seven weeks before admission the tumor was tapped, but rapidly refilled. In February 1865, under chloroform, an incision was made disclosing several adhesions between the peritoneum and the front and sides of the tumor, which were divided by actual cautery. The tumor was then tapped and sixteen pints of fluid removed, when it was found to be further adherent to the left iliac crest, to several inches of the sigmoid flexure, to the meso-rectum, and bladder. These adnesions were divided by actual cautery as far as practicable, and in all twelve patches were seared. One bleeding vessel, however, deep down between the uterus and rectum which could not be reached either by actual cautery or for the purpose of ligature, was suffered to remain patent, partly in consequence of the severe loss of blood already undergone and the consequent exhausted state of the patient, and partly because it had ceased to bleed before the operation was completed. The pedicle having been also divided by actual cautery was then returned to the pelvic cavity and the wound closed by silver sutures. The patient died twenty seven hours after the operation, exhibiting symptoms of internal hemorrhage during the last five hours. On careful post mortem examination, the pelvis was found to contain about a pint of nearly pure blood, the source of which was traced to the site of adhesion to the meso-rectum, to which it was impracticable to apply the hot iron. Signs of commencing peritonitis were also present. As this was the first case in which we had an opportunity of making a post mortem examination after the pedicle and adhesions had been divided by actual cautery, it is of great practical importance to observe that no one spot so divided had given way, but that the hemorrhage was entirely from the spot not cauterized.

Case No. 2. A. M., aet 45, single. A needlewoman, who, to use her own words, "had to spend more money on lodgings than food, as she could get no work unless she lived in respectable lodgings." Swelling in abdomen first noticed two and a half years ago. Catamenia regular. On March 16th, 1865, under chloroform, a primary incision of five inches was made. The adhesions were few and easily broken. Ten pints of fluid having been drawn off, the tumor was withdrawn and the pedicle divided by actual cautery, and the wound closed. A low form of peritonitis set in forty-eight hours after operation which speedily carried her off, ninety hours after the removal of the tumor. On post mortem examination a very imperfect attempt at union of the lower part of the incision was found. Intestines were inflated with gas, and matted together by recent lymph. A few ounces of serum were in the abdominal cavity, *but the seared pedicle was perfectly free from any symptom of secondary hemorrhage.* The heart was hypertrophied, the aortic valves thickened and puckered; other organs healthy.

CASE No. 3. M. H., aet 55, married. Has ceased to menstruate for four years. Eighteen months ago first noticed an enlargement in abdomen. Chloroform was administered July 19th, 1866, and an incision of six inches made. No adhesions were found anteriorly, but four pints of fluid, opaque and milky, were evacuated from the abdominal cavity. The tumor was extracted, and a small adhesion of the omentum exposed. This was separated, and the clamp applied to the pedicle, *but through failure of the cautery two large arteries were tied by twine ligature.* There was cystic disease of the omentum, two large layers of which were separated and formed a cavity containing transparent fluid which was evacuated. Low peritonitis ensued to which the patient succumbed the sixth day after operation. The post mortem examination revealed lack of union in the incision, general peritonitis with yellowish lymph, and universal slight adhesions. *No blood had escaped from the seared surface of the pedicle which was not included in the ligature.*

These cases, sir, are to me most satisfactory evidence of the value of the actual cautery in treating the pedicle, and when to these, one adds the large proportion of recoveries from operation after this method, I think I may safely commend it to my brother surgeons. In conclusion, sir, allow me to suggest to the profession that it would be even an advance on this treatment if we could show that the galvanic cautery could be safely substituted for the actual cautery.

